# Neoadjuvant Chemotherapy in Patients with HER2-Negative Breast Cancer: A Report from Clinical Breast Cancer Registry of Iran

**DOI:** 10.34172/aim.2024.30

**Published:** 2024-04-01

**Authors:** Kamran Roudini, Mehrzad Mirzania, Tahereh Yavari, Monireh Sadat Seyyedsalehi, Azin Nahvijou, Jayran Zebardast, Mina Saadat, Ahmad Khajeh-Mehrizi

**Affiliations:** ^1^Department of Hematology and Medical Oncology, Cancer Institute, Imam Khomeini Hospital Complex, Tehran University of Medical Sciences, Tehran, Iran; ^2^Department of Internal Medicine, Shariati Hospital, Tehran University of Medical Sciences, Tehran, Iran; ^3^Cancer Research Center, Cancer Institute, Tehran University of Medical Sciences, Tehran, Iran; ^4^Department of Medical and Surgical Sciences, University of Bologna, Bologna, Italy; ^5^Department of Cognitive Linguistics, Institute for Cognitive Science Studies (ICSS), Tehran, Iran; ^6^Advanced Diagnostic and Interventional Radiology Research Center (ADIR), Tehran University of Medical Science, Tehran, Iran; ^7^Student Research Committee, School of Nursing and Midwifery, Shahroud University of Medical Science, Shahroud, Iran

**Keywords:** Breast cancer, HER2-negative, Pathologic complete response, Recurrence, Survival

## Abstract

**Background:**

Neoadjuvant chemotherapy (NCT) has become an increasingly popular approach in management of breast cancer (BC). This study was conducted to evaluate the pathologic response and 36-month recurrence and survival rates of patients with human epidermal growth factor receptor 2 (HER2)-negative BC treated with different NCT regimens.

**Methods:**

A total of 163 female patients with HER2-negative BC who received NCT during 2017-2020 were identified from the Clinical Breast Cancer Registry of Iran and entered the study. The prescribed NCT regimens included 4 cycles of doxorubicin plus cyclophosphamide, 4 cycles of doxorubicin plus cyclophosphamide followed by 4 cycles of paclitaxel, 4 cycles of doxorubicin plus cyclophosphamide followed by 4 cycles of docetaxel or 6 cycles of doxorubicin plus cyclophosphamide plus docetaxel (TAC).

**Results:**

Thirty-two patients (19.6%) experienced pathologic complete response (pCR). TAC regimen, triple negative-BC and ki67>10% were significantly associated with increased pCR. The recurrence, overall survival (OS) and disease-free survival (DFS) rate at 36 months for all patients were 16.6%, 84.7% and 79.8%, respectively. Type of neoadjuvant regimen as well as age, hormone receptor status, Ki67, grade, clinical stage, type of surgery and pathologic response to chemotherapy did not significantly influence the survival and recurrence; however, TAC results in improved recurrence, OS and DFS rates.

**Conclusion:**

This study provides further evidence that NCT is a viable treatment option for patients with HER2-negative BC. The TAC regimen resulted in a significantly higher pCR rate compared to other regimens, but did not result in a significant improvement in recurrence, OS and DFS and rates.

## Introduction

 Breast cancer (BC) is the most common malignancy in women.^1^ Globally, BC accounts for 25% of all types of cancers including 1.7 million new cases per year.^2^ It is a complex and heterogeneous disease, and its treatment often involves a combination of surgery, radiation therapy, and systemic therapy.^3,4^ The treatment of BC can have a significant impact on the patient’s quality of life.^5^ Recent efforts to provide new treatments for BC have focused on targeted therapies, immunotherapy, and combination therapies, as well as the development of novel drug delivery systems.^6-8^

 Neoadjuvant chemotherapy (NCT), commonly prescribed before a surgery, has become an increasingly popular approach for treating BC.^9,10^ It allows for the evaluation of tumor response to chemotherapy and may improve the chances of breast-conserving surgery.^11-14^ Also, NCT affects the tumor microenvironment and immune response reflecting the potential for the development of novel treatment strategies.^15^ It has been reported that NCT provides the opportunity for a more individualized approach to treatment and can lead to improved outcomes, particularly in patients with locally advanced or inflammatory BC.^16,17^

 Various medications are prescribed for NCT of BC, including anthracyclines, taxanes, and platinum-based agents.^18^ Platinum-based drugs, such as cisplatin and carboplatin, inhibit DNA synthesis and ultimately lead to apoptosis.^19^ Anthracyclines, such as doxorubicin and epirubicin, are potent cytotoxic agents that interfere with DNA replication and induce cell death.^20^ Taxanes, such as paclitaxel and docetaxel, exert their anti-cancer effects by stabilizing microtubules and disrupting the normal cell division process.^21^ To optimize treatment outcomes, these agents are often used together or alongside targeted therapies, like human epidermal growth factor receptor 2 (HER2) inhibitors or cyclin-dependent kinase 4 and 6 (CDK 4/6) Inhibitors.^22^ Tumor size, Ki67 percent, HER2, hormone (estrogen and progesterone) receptor, and overall health status are considered in the selection of specific chemotherapeutic agents and dosing regimens.^23^ Ki67 percent is a measure of cancer cell proliferation and can predict how cancer cells will respond to certain treatments.^3^ HER2 inhibitors should be used in conjunction with chemotherapy for BC with HER2 amplification, while BC without HER2 amplification (HER2 negative) does not require these agents.^4^ HER2 negative BC can be classified by the presence of hormone receptors, and those without hormone receptors are called triple-negative breast cancers (TNBCs) which tend to be more aggressive and have fewer targeted treatment options compared to other BC subtypes.^23^

 Meanwhile, the use of NCT for BC treatment has been a subject of debate, with conflicting reports regarding the effects on patient outcomes and survival.^24,25^ Some studies have demonstrated that NCT can result in significant tumor shrinkage, allowing for less invasive surgical interventions and improved patient outcomes.^26,27^ However, other studies have suggested that the use of NCT does not provide significant survival benefits over traditional adjuvant chemotherapy (ACT).^24,28^ Moreover, concerns have been raised about the potential for NCT to increase the risk of disease recurrence, particularly in patients with TNBC.^24,29,30^ Studies have shown that various NCT regimens have different effects on the response rate of patients with BC.^25,31,32^

 Despite these controversies, NCT remains an important treatment option for BC, and ongoing research is focused on identifying patient subgroups who may benefit the most from this approach.^24^ This study was conducted to evaluate the three-year survival of patients with HER2 negative BC who underwent NCT. We also compared the pathologic response rate, recurrence and mortality among patients treated with different NCT regimens.

## Materials and Methods

 All patients newly diagnosed with BC during 2017–2020 and treated with NCT were identified from the Clinical Breast Cancer Registry of Iran.^33,34^ Female patients aged 18 or more with HER2-negative invasive ductal BC who received NCT were considered for inclusion in this study. Patients with a prior or synchronous other malignancies and those who were lost to follow-up after surgery were excluded from the study.

 The following data were obtained from the registry: age, Ki67%, grade, hormone receptor status, clinical stage, type of surgical therapy, NCT regimen, pathologic response, recurrence and survival status. Neoadjuvant regimens that were prescribed were as follows: (1) 4 cycles of doxorubicin plus cyclophosphamide (AC), (2) 4 cycles of doxorubicin plus cyclophosphamide followed by 4 cycles of paclitaxel (ACP), (3) 4 cycles of doxorubicin plus cyclophosphamide followed by 4 cycles of docetaxel (ACD), (4) 6 cycles of doxorubicin plus cyclophosphamide plus docetaxel (TAC). Response to NCT in surgical specimens were categorized into complete, partial and no response. Pathologic complete response (pCR) was defined as complete absence of viable tumor in the specimen. Pathologic partial response (pPR) and pathologic non-response (pNR) were defined as less than 50% and more than 50% of the treated tumor occupied by viable tumor cells, respectively.

###  Statistical Analysis

 Categorical variables are presented as number (%) whereas continuous variables are shown as mean (standard deviation). Using Fisher’s exact and Chi-square tests, we assessed patient characteristics and pathologic response in relation to neoadjuvant regimens. Survival analyses were made using the Kaplan–Meier method and compared by the log-rank test. Overall survival (OS) was defined as the time from cancer diagnosis until death from any cause. Disease-free survival (DFS) was defined as the period between the date of cancer diagnosis and the date of disease recurrence or death from any cause. The Cox proportional hazards regression model was used for computing hazard ratios (HRs) and multivariate survival analysis. The cumulative incidence of recurrence rates was estimated using competing risk methods and compared by the Gray test. Estimated HRs were calculated by Fine and Gray regression modeling to evaluate effects of variables on the risk of recurrence. *P* values less than 0.05 were considered to be statistically significant. SPSS Statistics for Windows (version 26.0; Armonk, NY: IBM Corporation) and R software (version 4.3.1; R Core Team, Vienna, Austria) were employed for statistical analyses.

## Results

###  Patient Characteristics

 A total of 163 patients were entered into analysis. The mean age of patients at the time of diagnosis was 48.5 years. Fifteen patients (9.2%) had TNBC while the tumors of 148 patients (91.8%) were hormone-receptor positive. The characteristics of the patients are listed in Table 1. Thirty patients (18.4%) received AC regimen while 61 (37.4%), 53 (32.5%) and 19 (11.7%) patients received ACD, ACP and TAC regimens, respectively.

**Table 1:** Characteristics of Patients

**Characteristic**		**TAC (n=19)**	**ACP (n=53)**	**ACD (n=61)**	**AC (n=30)**	***P*** ** Value**
Age	≤ 40	3 (6.7%)	14 (31.1%)	14 (31.1%)	14 (31.1%)	0.14
	41-50	10 (20.4%)	16 (32.7%)	17 (34.7%)	6 (12.2%)	
	51-60	4 (8.9%)	16 (35.6%)	21 (46.7%)	4 (8.9%)	
	≥ 61	2 (8.3%)	7 (29.2%)	9 (37.5%)	6 (25%)	
Hormone receptor	Positive	17 (11.5%)	48 (32.4%)	56 (37.8%)	27 (18.2%)	0.98
	Negative	2 (13.3%)	5 (33.3%)	5 (33.3%)	3 (20%)	
Ki67	≤ 10%	4 (9.1%)	15 (34.1%)	14 (31.8%)	11 (25%)	0.15
	> 10%	14 (13.6%)	32 (31.1%)	45 (43.7%)	12 (11.7%)	
	unknown	1 (6.3%)	6 (37.5%)	2 (12.5%)	7 (43.8%)	
Grade	1	2 (10%)	3 (15%)	10 (50%)	5 (25%)	0.60
	2	12 (12.6%)	34 (35.8%)	34 (35.8%)	15 (15.8%)	
	3	5 (10.4%)	16 (33.3%)	17 (35.4%)	10 (20.8%)	
Clinical stage	IIA	5 (8.6%)	17 (29.3%)	21 (36.2%)	15 (25.9%)	0.69
	IIB	6 (15%)	10 (25%)	18 (45%)	6 (15%)	
	IIIA	1 (6.7%)	6 (40%)	5 (33.3%)	3 (20%)	
	IIIB	3 (10%)	11 (36.7%)	12 (40%)	4 (13.3%)	
	IIIC	4 (20%)	9 (45%)	5 (25%)	2 (10%)	
Surgical therapy	BCT	5 (14.3%)	12 (34.3%)	12 (34.3%)	6 (17.1%)	0.92
	Mastectomy	14 (10.9%)	41 (32%)	49 (38.3%)	24 (18.8%)	

TAC, docetaxel + doxorubicin + cyclophosphamide; ACP, doxorubicin + cyclophosphamide + paclitaxel; ACD, doxorubicin + cyclophosphamide + docetaxel; AC, doxorubicin + cyclophosphamide; BCT, Breast-conserving therapy.

###  Response to Neoadjuvant Chemotherapy 

 Thirty-two patients (19.6%) experienced pCR compared with 131 patients(80.4%) who had residual disease in their surgical specimen. Significant increased pCR rates were observed for patients treated with TAC compared with another regimen (*P* < 0.001). In terms of pathologic response based on the neoadjuvant regimen (Table 2), patients treated with TAC showed pCR of 42.1%, pPR of 52.6%, and negligible pNR rate of 5.3%. This contrasts with the other regimens, for instance, ACP which had 18.9% pCR, 62.3% pPR, and 18.9% pNR. Patients with TNBC experienced significantly higher pCR than those with hormone receptor-positive cancer (*P* = 0.007). Within the subset of hormone receptor status, positive patients had pCR of 16.9%, pPR of 56.8%, and pNR of 26.4% while negative ones demonstrated pCR of 46.7%, pPR of 20%, and pNR of 33.3% (Table 2). Significantly lower pCR rates were found in patients with Ki67 ≤ 10% versus those with Ki67 > 10% (*P* = 0.02). Specifically, patients with Ki67 ≤ 10% exhibited pCR of 9.1%, pPR of 70.5%, and pNR of 20.5%, while those with Ki67 > 10% had pCR of 27.2%, pPR of 49.5%, and pNR of 23.3% (Table 2). Grade and clinical stage did not have any significant effect on pathologic response, although it has been observed that patients with higher stage and clinical stage tend to experience better response to NCT (Table 2).

**Table 2:** Pathologic Response Based on Neoadjuvant Regimen and Patient Characteristics.

		**pCR**	**pPR**	**pNR**	***P*** ** value**
Neoadjuvant regimen	TAC	8 (42.1%)	10 (52.6%)	1 (5.3%)	< 0.001
	ACP	10 (18.9%)	33 (62.3%)	10 (18.9%)	
	ACD	12 (19.7%)	39 (63.9%)	10 (16.4%)	
	AC	2 (6.7%)	5 (16.7%)	23 (76.7%)	
Hormone receptor	positive	25 (16.9%)	84 (56.8%)	39 (26.4%)	0.007
	negative	7 (46.7%)	3 (20%)	5 (33.3%)	
Ki67	≤ 10%	4 (9.1%)	31 (70.5%)	9 (20.5%)	0.02
	> 10%	28 (27.2%)	51 (49.5%)	24 (23.3%)	
Grade	1	3 (15%)	9 (45%)	8 (40%)	0.14
	2	14 (14.7%)	56 (58.9%)	25 (26.3%)	
	3	11 (22.9%)	23 (47.9%)	14 (29.2%)	
Clinical Stage	IIA	10 (17.2%)	26 (44.8%)	22 (37.9%)	0.33
	IIB	8 (20%)	26 (65%)	6 (15%)	
	IIIA	3 (20%)	7 (46.7%)	5 (33.3%)	
	IIIB	5 (16.7%)	18 (60%)	7 (23.3%)	
	IIIC	6 (30%)	10 (50%)	4 (20%)	

pCR, pathologic complete response; pPR, pathologic partial response; pNR, pathologic no response; TAC, docetaxel + doxorubicin + cyclophosphamide; ACP, doxorubicin + cyclophosphamide + paclitaxel; ACD, doxorubicin + cyclophosphamide + docetaxel; AC, doxorubicin + cyclophosphamide.

 To underscore the distinctions between the regimens, patients treated with TAC exhibited significantly higher overall response rates, reaching 94%, compared to those on other regimens, with ACP at 79%, ACD at 88%, and AC at 80% (*P* < 0.001). Such stark contrasts highlight the potential superiority of TAC in eliciting favorable responses, a finding that warrants further discussion and analysis.

###  Survival 

**Figure 1:**
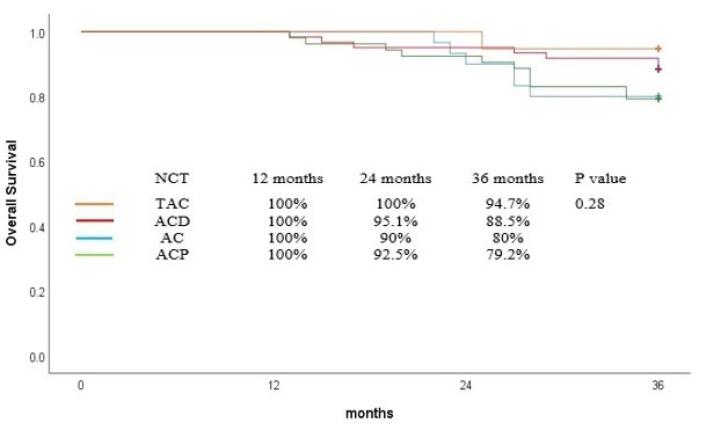
Overall Survival Curve for Patients Based on Neoadjuvant Chemotherapy

 The OS rate at 36 months was 84.7% for all patients and was comparable among the patients who received various neoadjuvant regimen (*P* = 0.28, Figure 1). However, TAC results in superior 36-month OS compared with other regimens (TAC = 94%, ACP = 79%, ACD = 88%, AC = 80%). Cox proportional regression revealed that no variable significantly influences the OS (Table 3).

**Table 3:** Univariable Analysis for Overall Survival, Disease-free Survival and Recurrence

		**OS**		**DFS**		**Recurrence**	
		**HR (95%CI)**	***P***	**HR (95%CI)**	***P***	**HR (95%CI)**	***P***
Neoadjuvant regimen	ACP vs. TAC	4.2 (0.54-32.8)	0.16	5.9 (0.78-45.1)	0.08	3.32 (0.38-28.4)	0.27
	ACD vs. TAC	4.2 (0.27-18.1)	0.45	3.5 (0.46-27.6)	0.22	2.89 (0.36-22.8)	0.31
	AC vs. TAC	4.09 (0.49-34)	0.19	4.05 (0.48-33.7)	0.19	4.53 (0.59-34.9)	0.14
Age	≤ 40 vs. ≥ 61	0.63 (0.19-2.07)	0.44	0.93 (0.31-2.77)	0.89	2.52 (0.54-11.6)	0.23
	41-50 vs. ≥ 61	0.69 (0.22-2.1)	0.52	0.94 (0.32-2.77)	0.92	2.56 (0.56-11.7)	0.22
	51-60 vs. ≥ 61	0.76 (0.24-2.42)	0.65	0.94 (0.31-2.82)	0.92	1.62 (0.32-8.04)	0.55
Hormone receptor	Negative vs. positive	1.35 (0.4-4.52)	0.62	1.63 (0.39-6.83)	0.5	1.28 (0.3-5.43)	0.73
Ki67	≤ 10% vs. > 10%	0.76 (0.31-1.81)	0.53	0.97 (0.44-2.11)	0.94	0.86 (0.36-2.05)	0.74
Grade	1 vs. 3	0.68 (0.14- 3.3)	0.63	0.91 (0.24-3.45)	0.89	0.81 (0.16-4.02)	0.79
	2 vs. 3	1.21 (0.5-2.96)	0.66	1.46 (0.65-3.29)	0.35	1.71 (0.68-4.28)	0.25
Clinical Stage	IIA vs. IIIC	3.74 (0.47-29.2)	0.2	2.22 (0.49-9.91)	0.29	2.22 (0.49-9.94)	0.29
	IIB vs. IIIC	3.89 (0.47-31.6)	0.2	2.81 (0.61-12.8)	0.18	1.88 (0.39-9.04)	0.43
	IIIA vs. IIIC	4.58 (0.47-44)	0.18	2.9 (0.53-15.8)	0.21	1.34 (0.18-9.51)	0.77
	IIIB vs. IIIC	2.77 (0.31-24.8)	0.36	1.8 (0.35-9.29)	0.48	1.39 (0.25-7.62)	0.70
Surgical therapy	BCT vs. Mastectomy	2.06 (0.61-6.89)	0.23	1.62 (0.62-4.21)	0.31	1.67 (0.57-4.83)	0.34
Pathologic response	pCR vs. pNR	0.22 (0.5-1.02)	0.05	0.65 (0.19-2.18)	0.49	0.55 (0.14-2.16)	0.39
	pPR vs. pNR	0.53 (0.23-1.2)	0.13	1.35 (0.6-3.06)	0.46	1.23 (0.51-2.97)	0.64

OS, overall survival; DFS, disease-free survival; HR, hazard ratio; CI, confidence interval; TAC, docetaxel + doxorubicin + cyclophosphamide; ACP, doxorubicin + cyclophosphamide + paclitaxel; ACD, doxorubicin + cyclophosphamide + docetaxel; AC, doxorubicin + cyclophosphamide; BCT, Breast-conserving therapy; pCR, pathologic complete response; pPR, pathologic partial response; pNR, pathologic no response. Univariable Cox proportional hazards model for PFS and OS. Univariable Fine-Gray hazards model for cumulative incidence of recurrence.

**Figure 2:**
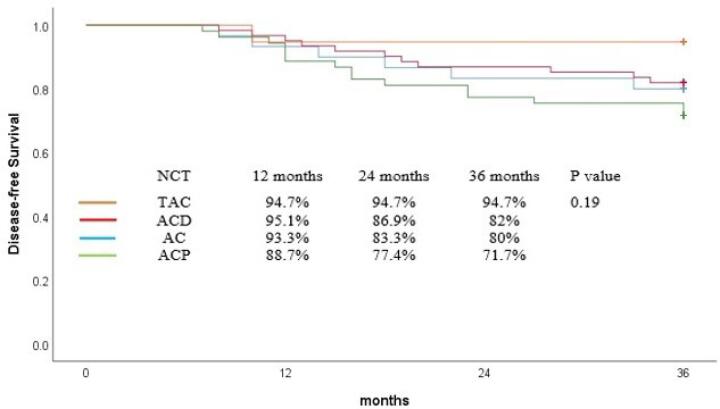
Disease-Free Survival Curve for Patients Based on Neoadjuvant Chemotherapy

 The DFS rate at 36 months was 79.8% for all patients. Similarly, TAC regimen insignificantly improved DFS in comparison with others and 36-month DFS was comparable among the patients who received different neoadjuvant regimens (TAC = 94%, ACP = 71%, ACD = 82%, AC = 80%, *P* = 0.19, Figure 2). In regression analyses, no variable significantly affected the DFS (Table 3).

 Multivariable regression analysis was done to determine the potential predictors of OS and DFS. Analysis including type of neoadjuvant regimen, age, hormone receptor status, Ki67, grade, clinical stage, type of surgery and pathologic response to chemotherapy demonstrated that no variable was a statistically significant prognostic factor affecting OS and DFS (Table 4).

**Table 4:** Multivariable Analysis for Overall Survival, Disease-free Survival and Recurrence

		**OS**		**DFS**		**Recurrence **	
		**HR (95% CI)**	***P***	**HR (95% CI)**	***P***	**HR (95% CI)**	***P***
Neoadjuvant regimen	ACP vs. TAC	3.49 (0.42-28.5)	0.24	5.39 (0.69-41.8)	0.10	4.39 (0.54-35.2)	0.16
	ACD vs. TAC	1.64 (0.19-13.7)	0.64	2.97 (0.37-23.4)	0.30	2.68 (0.32-22.05)	0.35
	AC vs. TAC	1.37 (0.12-15.1)	0.19	4.37 (0.45-42.4)	0.20	4.51 (0.45-44.63)	0.19
Age	≤ 40 vs. ≥ 61	1.05 (0.28-3.97)	0.93	1.10 (0.35-3.44)	0.87	3.33 (0.68-16.20)	0.13
	41-50 vs. ≥ 61	1 (0.25-3.92)	0.99	0.96 (0.30-3.09)	0.95	3.02 (0.60-15.04)	0.17
	51-60 vs. ≥ 61	0.85 (0.21-3.50)	0.83	0.79 (0.23-2.70)	0.71	1.76 (0.32-9.60)	0.51
Hormone receptor	Negative vs. positive	1.06 (0.19-5.66)	0.94	0.84 (0.18-4)	0.83	1.01 (0.21-4.85)	0.98
Ki67	≤ 10% vs. > 10%	0.61 (0.21-1.80)	0.37	1.08 (0.45-2.62)	0.85	0.71 (0.26-2.33)	0.69
Grade	1 vs. 3	1.28 (0.23- 7.20)	0.77	1.13 (0.28-4.56)	0.85	1.04 (0.20-5.45)	0.95
	2 vs. 3	1.35 (0.42-4.32)	0.60	1.24 (0.47-3.24)	0.65	1.68 (0.59-4.73)	0.32
Clinical Stage	IIA vs. IIIC	2.56 (0.29-22.04)	0.39	1.82 (0.39-8.43)	0.44	1.57 (0.33-7.41)	0.56
	IIB vs. IIIC	4.1 (0.45-37.04)	0.20	2.23 (0.46-10.6)	0.31	1.29 (0.25-6.56)	0.75
	IIIA vs. IIIC	3.08 (0.22-42.65)	0.40	2.13 (0.32-14.08)	0.43	1.27 (0.15-10.16)	0.82
	IIIB vs. IIIC	2.55 (0.26-24.13)	0.31	1.58 (0.29-8.44)	0.59	1.29 (0.22-7.47)	0.77
Surgical therapy	BCT vs. Mastectomy	1.54 (0.41-5.72)	0.51	1.34 (0.48-3.71)	0.56	1.77 (0.58-5.44)	0.31
Pathologic response	pCR vs. pNR	0.21 (0.04-1.17)	0.07	0.80 (0.19-3.39)	0.77	0.53 (0.11-2.46)	0.42
	pPR vs. pNR	0.40 (0.12-1.30)	0.13	1.52 (0.49-4.66)	0.46	1.21 (0.39-3.70)	0.73

OS, overall survival; DFS, disease-free survival; HR, hazard ratio; CI, confidence interval; TAC, docetaxel + doxorubicin + cyclophosphamide; ACP, doxorubicin + cyclophosphamide + paclitaxel; ACD, doxorubicin + cyclophosphamide + docetaxel; AC, doxorubicin + cyclophosphamide; BCT, Breast-conserving therapy; pCR, pathologic complete response; pPR, pathologic partial response; pNR, pathologic no response. Multivariable Cox proportional hazards model for PFS and OS. Multivariable Fine-Gray hazards model for cumulative incidence of recurrence.

 While the OS and DFS rates were generally high across all regimens, a nuanced look at the data reveals TAC’s potential edge. At 36 months, TAC achieved 94% OS and 94% DFS, outstripping ACP (79% OS, 71% DFS), ACD (88% OS, 82% DFS), and AC (80% OS, 80% DFS), although the differences were not statistically significant (OS, *P* = 0.28; DFS, *P* = 0.19). These regimen-specific outcomes illuminate the varying impacts of the treatment regimens on patient survival, underscoring the need for a tailored approach to regimen selection.

###  Recurrence 

**Figure 3:**
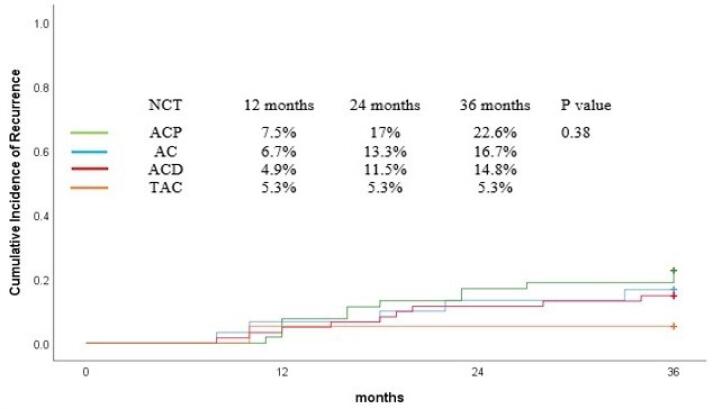
Cumulative Incidence of Recurrence Curve for Patients Based on Neoadjuvant Chemotherapy

 The recurrence rate at 36 months was 16.6% for all patients. Figure 3 shows the cumulative incidence of recurrence. There was no significant difference in the 36-month cumulative incidence rates of recurrence among patients who took different neoadjuvant regimens (TAC = 5.3%, ACP = 22.6%, ACD = 14.8%, AC = 16.7%, *P* = 0.38). No variable was found in univariable and multivariable analysis that significantly influenced the recurrence rate (Tables 3 and 4).

## Discussion

 We conducted this study to evaluate the three-year survival of patients with BC who received one of four regimens of NCT and compare the outcomes of treatment among the regimens. We investigated the effect of tumor characteristics, including hormone receptor status, Ki67, grade, and clinical stage on treatment outcomes. There was no significant difference in OS and DFS rates among patients who received different NCT regimens. Meanwhile, TAC tended to result in superior OS and DFS compared with other regimens. Overall, the results showed that TAC regimen and TNBC were more associated with pCR, while patients with Ki67 ≤ 10% were less likely to achieve pCR. Although not statistically significant, patients with higher tumor grade and clinical stage seemed to exhibit a better response to NCT. These findings are consistent with previous studies that have reported the association between these factors and treatment outcomes in BC patients treated with NCT.

 Delving deeper into the effects of the NCT regimens, our data suggest a promising trend for the TAC regimen, demonstrating higher pCR, OS, and DFS rates compared to other regimens. Although these differences did not reach statistical significance, the consistency of TAC’s performance across multiple outcome measures cannot be overlooked. The pronounced disparity in overall response rates, with TAC at 94% compared to 79% for ACP, 88% for ACD, and 80% for AC, points to its potential as a more efficacious option for inducing pathologic response. Similarly, the survival analysis revealed TAC’s superior performance, achieving 94% OS and 94% DFS at 36 months, a trend that held even when adjusting for various patient and tumor characteristics.

 Early diagnosis and chemotherapy have improved the survival of patients with BC, although morbidities caused by chemotherapy may affect patients in the long term.^35^ Our investigation showed that NCT resulted in a favorable three-year OS rate of 84.7% and DFS rate of 79.8%. Most of the pCR in our study were from the TAC group. Combinations of docetaxel, doxorubicin, and cyclophosphamide have been used with favorable clinical and pathological results for treating BC. In a recent study, researchers explored the efficacy and safety of pyrotinib plus TAC for HER2 + BC.^36^ Overall, 37.0% of patients achieved total pCR, 37.0% pCR in the breast, and 85.2% pCR in lymph node. In a trial of TAC regimen on 45 patients with BC, authors reported a clinical response rate of 59% (95% CI 42% to 73%) within the breast and overall (breast and axilla) response rate of 49% (95% CI 38% to 72%) in the intention-to-treat population.^37^ The pCR was 10% in the breast, 27% in the axillary lymph nodes, and 7% in both. They also reported a 5-year survival rate of 80%. Another trial resulted in 16% pCR for a TAC group.^38^ Overall, TAC was a recommended regimen for BC in the literature. However, the discrepancies may be due to differences in patient populations, treatment protocols, or study designs, implying the need for further research to find the optimal NCT regimens for different patient subgroups.

 A linear relationship has been suggested between increasing pCR rate and increasing recurrence-free survival.^39^ A meta-analysis by Cortazar et al included 11 955 patients from 12 randomized clinical trials and showed that NAC significantly improved the pCR rate and event-free survival compared with ACT.^40^ Meanwhile, the association between pCR and long-term outcomes was strongest in patients with aggressive tumor subtypes, such as TNBC and HER2-positive, and hormone receptor-negative tumors. In TNBC, the association between pCR and long-term outcomes was strongest with HR of 0.24 (95%CI 0.18-0.33) for event-free survival and 0.16 (0.11-0.25) for OS. Broadly speaking, TNBC is a cancer that exhibits a significant degree of heterogeneity and mutations as well as abnormal activation of signaling pathways. Recent studies suggested that targeted therapies are more promising treatment options against TNBC.^6^ Other studies have also reported that the attainment of a pCR after NCT in TNBC patients leads to improved survival.^19,32^ In a meta-analysis, Xia et al compared NCT and ACT in TNBC patients. They included nine studies with a total of 36,480 patients, where 29.41% received NCT, and 70.59% received ACT. The results showed that NCT with pCR significantly improved OS and DFS. They suggested that NCT with pCR is superior to ACT in improving survival outcomes for TNBC patients.^32^

 Commonly, Ki67 has been proposed as a useful clinical marker for BC subtype classification, prognosis, and prediction of therapeutic response.^41^ A recent review study conducted by Zhang et al assessed the role of Ki67 in NCT therapy for BC.^41^ They concluded that NCT is the first choice for TNBC and HER2-positive BC. However, because of the uniformly low rate of pCR and slow response, NCT was not suggested as the preferred option for rapidly reducing the stage of large tumor burdens.^40-42^ They also reported that higher pretreatment Ki67 was more likely to attain pCR after NCT and that higher pretreatment Ki67 may improve the prognostic significance of clinical response in NCT.^43-45^ Our study showed that patients with TNBC experienced significantly higher pCR than those with hormone-receptor positive BC. This is consistent with a study by Colleoni et al who found a statistically significant higher pCR rate in patients with estrogen and progesterone absent tumors (adjusted OR = 14.4).^46^ Previous studies indicated that the absence of hormone receptor expression and Ki-67 ≥ 20% were predictive of a clinical complete response.^47^ A high tumor grade has been reported as a predictive factor of pCR.^47^ However, our results did not replicate the significant effect of tumor grade in pCR. Also, the current study did not find a significant difference in survival outcomes across patients treated with different NAC regimens. These require more delineation with a larger sample size and longer follow-up period.

 One potential reason for the absence of significant differences in OS and DFS across the different NCT regimens may be attributed to the inherent variability in patient and tumor characteristics, which have a profound influence on treatment outcomes. It is conceivable that while the chemotherapeutic agents themselves possess distinct mechanisms of action, the nuances in individual patient profiles, tumor biology, and disease staging could counterbalance these differences, leading to analogous survival rates. Furthermore, the intricacies of tumor microenvironment interactions, resistance mechanisms, and other yet unidentified biological variables might play a pivotal role in determining individual responses to treatment, thus nullifying any overt differences among the regimens. While our study did illuminate certain trends, such as TAC’s superior performance in terms of pCR, OS, and DFS rates, it is crucial to highlight the possible presence of confounding variables or biases that might have been inadvertently introduced during patient selection, treatment assignment, or data interpretation. A more comprehensive, prospective study with stratified randomization and longer follow-up might offer deeper insight into the precise reasons behind the observed equivalence in survival rates among the NCT regimens.

 In the groundbreaking IMpassion130 trial led by Schmid et al, the combined therapeutic efficacy of Atezolizumab and Nab-Paclitaxel was assessed in patients with metastatic TNBC. With their patient cohort having a median age of 55 years in comparison to the average age of 48.5 years in our study, the IMpassion130 trial reported an objective response rate of 53%, standing in contrast to our pCR rate of 19.6%. Furthermore, the IMpassion130 trial noted a median progression-free survival (PFS) of 7.2 months. This, when compared with our more extended DFS rate of 79.8% at 36 months and an OS rate of 84.7%, underscores the potential disparities in treatment outcomes based on therapeutic choices and patient cohorts.^48^

 In a study conducted by Yamamoto et al^49^ researchers explored the potential advantages of adjuvant capecitabine for HER2-negative BC patients who had residual disease following neoadjuvant treatment. The study’s participants presented a 5-year OS rate of 89.2%, aligning closely yet slightly exceeding our 36-month OS rate of 84.7%. Their DFS, reported at 74.1% over 5 years, also mirrors our 36-month statistic of 79.8%. Although the direct response rate in terms of pCR for the CREATE-X trial is not directly comparable, the similarities and contrasts in survival metrics between our research and theirs highlight the pivotal role of tailored therapeutic strategies, and the significance of deep-diving into specific patient and tumor characteristics in driving optimal care decisions.

 In the renowned NSABP B-27 trial orchestrated by the National Surgical Adjuvant Breast and Bowel Project, the potential of introducing docetaxel to a foundational regimen of AC in the neoadjuvant setting was closely examined for operable BC, inclusive of the HER2-negative subtype.^50^ Participants, who were subjected to varying sequences of the aforementioned drugs, presented a pCR rate of 26.1% when doxorubicin and cyclophosphamide were succeeded by docetaxel. This stands in contrast with our cohort, composed of patients with an average age of 48.5 years, which manifested a pCR rate of 19.6%. While the NSABP B-27 trial did not note pronounced disparities in OS between their arms at the five-year benchmark, our study highlighted an OS rate of 84.7% at 36 months. Similarly, the trial’s modest enhancement in DFS with the inclusion of docetaxel resonates with our compelling DFS rate of 79.8% over the same 36-month span. The juxtaposition of these investigations accentuates the intricate tapestry of neoadjuvant therapeutic strategies and underscores the import of regimen sequencing and drug combination in achieving optimal patient outcomes.

 In a significant undertaking by Kim et al, researchers embarked on contrasting the outcomes of neoadjuvant endocrine therapy (NET) with those of NCT in pre-menopausal patients diagnosed with ER-positive, HER2-negative, lymph node-positive BC.^51^ Sourced from seven hospitals in South Korea, the patients in their study, who underwent 24 weeks of either therapeutic regimen, painted a decisive picture: those under NCT displayed a markedly higher clinical response rate of 83.7% versus the 52.9% in the NET cohort. This discrepancy is pronounced when juxtaposed with our study, where participants (with an average age of 48.5 years) reported a pCR rate of 19.6%. Kim and colleagues also observed a marginally higher pCR in the NCT group (3.4%) compared to the NET group (1.2%). In comparison, our study flaunted a robust OS of 84.7% at 36 months and a DFS rate of 79.8%. The differential outcomes between the investigation by Kim et al and ours accentuate the therapeutic efficacy of individualized treatments, emphasizing the ever-evolving dynamics of BC care.

 In general, our findings are consistent with previous studies that have reported improved survival outcomes with NCT in patients with BC. For instance, in a trial of NCT for 72 BCs, mastectomy was avoided in 46% of patients, 42% converted to negative nodes after NCT, and 18% achieved a pCR.^13^ Five-year survival for patients with pCR was 100%, compared with 74% in the group with partial response and 48% in the group with no response or progression. Patients with the ER + /HER2 + subtype were most likely to have no response or progression during chemotherapy. Five-year survival was highest for patients achieving pCR. They concluded that NCT decreased the mastectomy rate, and reduced the need for axillary lymph node dissection. In a systematic review of 14 randomized trials with 5500 women, Mieog et al assessed the effectiveness of NCT versus ACT for early BC.^29^ They found that OS was equivalent in both groups, but the neoadjuvant group had fewer adverse effects. There was no survival difference between NCT and ACT (HR = 0.98, 95% CI = 0.87 to 1.09). It was concluded that NCT is an established treatment option for early BC. In a meta-analysis, Chen et al compared the survival benefits of NCT versus ACT for operable BC.^24^ The study reviewed 16 randomized clinical trials. Overall, 787 deaths were reported among 2794 patients assigned to NCT groups and 816 deaths among 2799 patients assigned to ACT groups. Subgroup analysis indicated that patients with pCR had better survival outcomes. The authors concluded that there was no significant difference in OS or recurrence-free survival between NCT and ACT groups.

 The relationship between treatment response and survival outcomes, particularly OS and DFS, has been a point of interest in oncological research. It is widely understood that achieving pCR often correlates with improved survival rates. In our study, while there was no significant difference in OS and DFS rates among different NCT regimens, a noticeable trend was observed where TAC exhibited higher pCR, OS, and DFS. This suggests a potential association between pCR achievement and favorable long-term outcomes. Specifically, the pronounced pCR rates achieved by the TAC regimen may be indicative of its superior ability to eradicate micrometastatic disease, thereby leading to improved OS and DFS rates. Furthermore, the trend of higher tumor grade and clinical stage of patients exhibiting a better response to NCT underscores the potential of these tumor characteristics to predict response to therapy. A positive treatment response not only suggests a reduction in the primary tumor but may also represent an effective systemic control, which subsequently leads to enhanced OS and DFS. However, it is essential to recognize that while pCR is a strong surrogate marker, other factors, including tumor biology and individual patient characteristics, play a pivotal role in long-term survival. Future studies should further explore this relationship to optimize treatment strategies based on individual patient profiles.

 One limitation of this study is its retrospective design, which may have led to selection bias. In addition, the follow-up period of three years may not have been sufficient to evaluate the long-term survival outcomes of patients with BC treated with NCT. Future prospective longitudinal studies with larger sample sizes and longer follow-up periods are needed to establish our findings and identify patient subgroups that may benefit the most from NCT.

## Conclusion

 In conclusion, this study provides further evidence that NCT is a viable treatment option for patients with BC, with favorable survival outcomes at least for three years. In summary, this study showed that that TAC regimen and TNBC were more associated with pCR, while patients with Ki67 ≤ 10% were less likely to achieve pCR. The TAC regimen resulted in a significantly higher pCR rate compared to other regimens, but did not result in a significant difference in recurrence, OS and DFS rates.
